# A case of Baraitser–Winter cerebrofrontofacial syndrome diagnosed by whole-exome sequencing

**DOI:** 10.1038/s41439-025-00319-x

**Published:** 2025-09-03

**Authors:** Kenichi Suga, Hiroki Sato, Masashi Suzue, Yukako Honma, Yasunobu Hayabuchi, Ryuji Nakagawa, Kayo Shinomiya, Nobuhiko Okamoto, Yuta Inoue, Naomi Tsuchida, Naomichi Matsumoto, Hiroyuki Morino, Yuishin Izumi, Maki Urushihara

**Affiliations:** 1 Department of Pediatrics, Tokushima University Hospital, Kuramotocho, Tokushima, Japan; 2 Department of Ophthalmology, Tokushima University Hospital, Kuramotocho, Tokushima, Japan; 3 Department of Medical Genetics, Osaka Women’s and Children’s Hospital, Izumi, Japan; 4 Department of Human Genetics, Yokohama City University Graduate School of Medicine, Yokohama, Japan; 5 Department of Rare Disease Genomics, Yokohama City University Hospital, Yokohama, Japan; 6 Department of Clinical Genetics, Yokohama City University Hospital, Yokohama, Japan; 7 Department of Medical Genetics, Tokushima University Hospital, Kuramotocho, Tokushima, Japan; 8 Department of Neurology, Tokushima University Hospital, Kuramotocho, Tokushima, Japan

**Keywords:** Paediatrics, Genetic testing

## Abstract

Here we report a heterozygous missense variant in the ACTB gene, NM_001101.5:c.209C>T (p.Pro70Leu), detected in a case of a mildly affected infant with Baraitser–Winter cerebrofrontofacial syndrome, characterized by unique craniofacial features, coloboma and mild developmental delay, but without lissencephaly. Baraitser–Winter cerebrofrontofacial syndrome cases with a similar mild phenotype have been reported to have the same variant in different populations, suggesting a genotype–phenotype correlation in this syndrome.

Baraitser–Winter cerebrofrontofacial syndrome (BWCFF, OMIM: #243310) is a rare congenital developmental disorder characterized by facial anomalies, coloboma, short stature and intellectual disability^1^. Distinctive facial features include hypertelorism, ptosis, high-arched eyebrows and a broad nasal tip and bridge. Most patients present with some degree of cortical dysplasia, pachygyria (often more marked in the frontal area) and subcortical band heterotopias. BWCFF can be definitively diagnosed only with exome sequencing identifying a gain-of-function variant in the *ACTB* (MIM*102630) or *ACTG1* genes (MIM*102560)^2,3^. *ACTB* gene encodes β-actin, which is essential for a number of cytoplasmic functions, including the regulation of cell shape and migration, as well as nuclear functions, such as regulation of gene expression, cell division and proliferation^3^.

A male newborn was delivered at 38 weeks of gestation to nonconsanguineous Japanese parents with no family history of note. Fetal ultrasound at 19 weeks of gestation revealed increased nuchal translucency and a horseshoe kidney. At birth, the infant displayed distinctive facial features, including a low nasal root, narrow palpebral fissures, low-set ears, micrognathia, telangiectasias and posterior neck edema (Fig. 1). He was admitted to the Growth Care Unit at Tokushima University Hospital, where he was placed in an incubator and received oxygen therapy and fluid management. A neonatal hearing screening test indicated a ‘refer’ result on the right side. An echocardiogram showed patent ductus arteriosus and mitral regurgitation, necessitating patent ductus arteriosus ligation at 1 month of age. Auditory brainstem response testing at 3 months of age confirmed severe right-sided hearing loss at 85 dB. Severe brachycephaly was noted at 5 months of age, but cranial suture premature synostosis was ruled out. Helmet therapy was initiated at that time. Developmentally, he achieved head control at 5 months of age, spoke his first word at 18 months of age and began walking at 20 months of age. At his most recent follow-up at 3 years and 5 months of age, he demonstrated contextually appropriate speech. His developmental quotient was 89 according to the Kinder Infant Development Scale. Brain magnetic resonance imaging performed at 1 year of age revealed mild frontal lobe atrophy and lateral ventricular dilation, with no evidence of pachygyria or subcortical band heterotopias. Ophthalmological evaluation revealed colobomas of the right iris, choroid and optic nerve (Fig. 1d). Echocardiography at 3 years of age revealed aortic valve stenosis with a peak velocity of 2.5 m/s, as well as mild-to-moderate mitral regurgitation. Chromosomal karyotyping showed 46,XY, and microarray-based chromosome analysis identified no pathological copy-number variants.

**Fig. 1:**
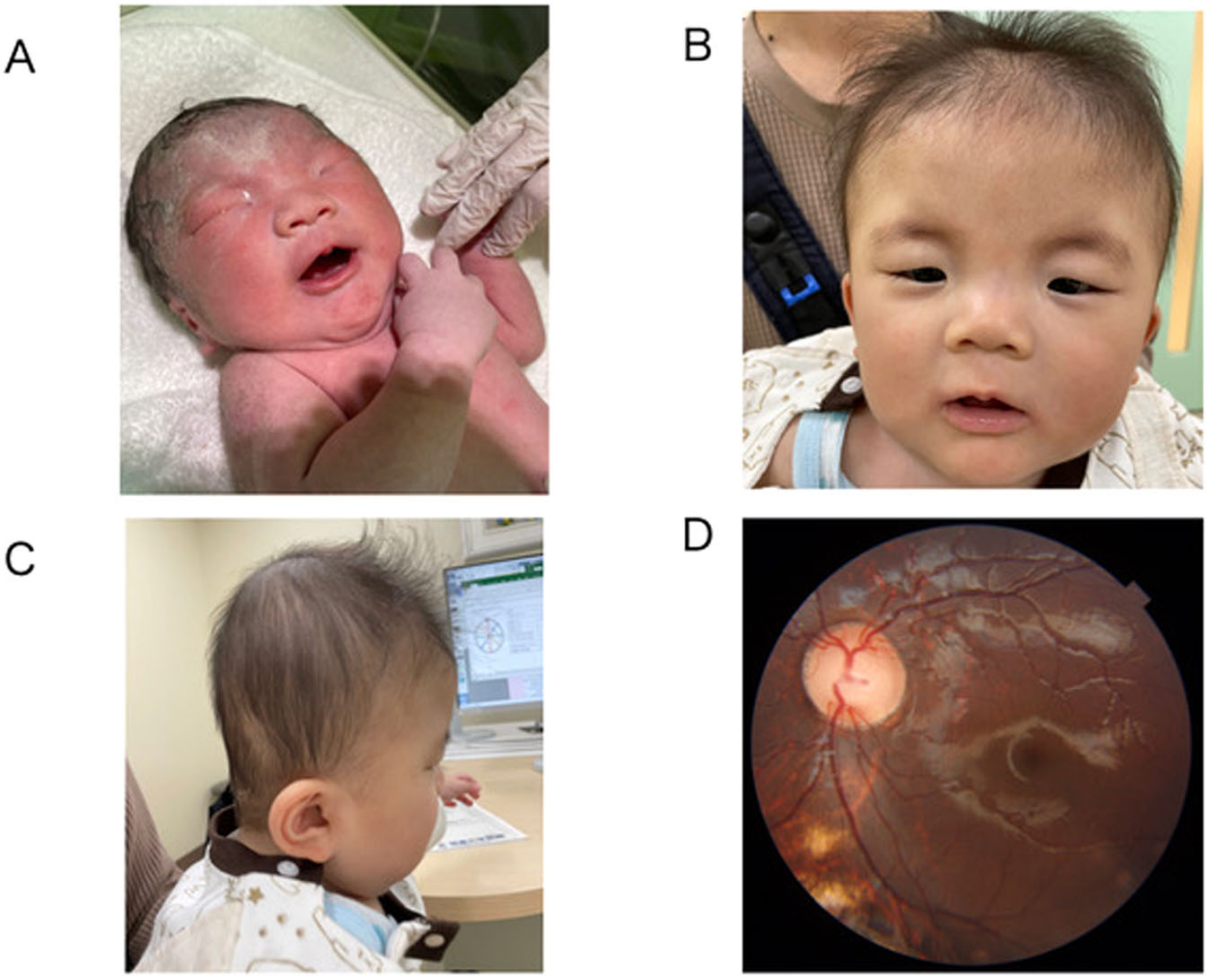
Clinical presenations of Baraitser-Winter cerebrofrontofacial syndrome. **A**–**C** Photographs of the patient at birth (**A**) and at 11 months of age (**B** and **C**), showing hypertelorism, arched eyebrows, ptosis, low-set ears, broad nasal bridge, low nasal root, long philtrum and thin upper lip. The lateral view (**C**) shows prominent brachycephaly. **D** Fundoscopy shows colobomas of the right choroid and optic nerve (yellow arrows).

Written informed consent for genetic testing was obtained from his parents. Whole-exome sequencing (WES) for the proband was performed as previously described^4^. Candidate variants identified from WES were validated by Sanger sequencing.

WES revealed a heterozygous missense variant [NM_001101.5:c.209C>T p.(Pro70Leu)] in the *ACTB* gene. Sanger sequencing confirmed the de novo variant in the patient (Fig. 2). This variant was not present in the gnomAD database. This variant has been reported in two cases of BWCFF^2,5^. In the ClinVar database, two reports described the same variant as ‘pathological’, and one further report described it as ‘likely pathological’. The altered amino acid is well conserved between species. This variant is located on the functional domain and has been reported to be one of the hotspots^6^. Pathological relevance was predicted using several protein function prediction tool (Sorting Intolerant From Tolerant (SIFT, http://sift.jcvi.org/), MutationTaster (http://MutationTaster.org/) and CADD (https://cadd.gs.washington.edu/)). According to the American College of Medical Genetics and Genomics and the Association for Molecular Pathology guideline, this variant was regarded as pathogenic (1 strong (PS1), 3 moderate (PM1, PM2 and PM6), 3 supporting (PP2, PP3 and PP5)). The clinical presentation aligned with the characteristics of BWCFF, leading to a final diagnosis of BWCFF. Genetic counseling was provided to the family, during which the results of the genetic testing were disclosed, and information about the natural course of BWCFF and necessary precautions was shared at 1 year 8 months of age.

**Fig. 2:**
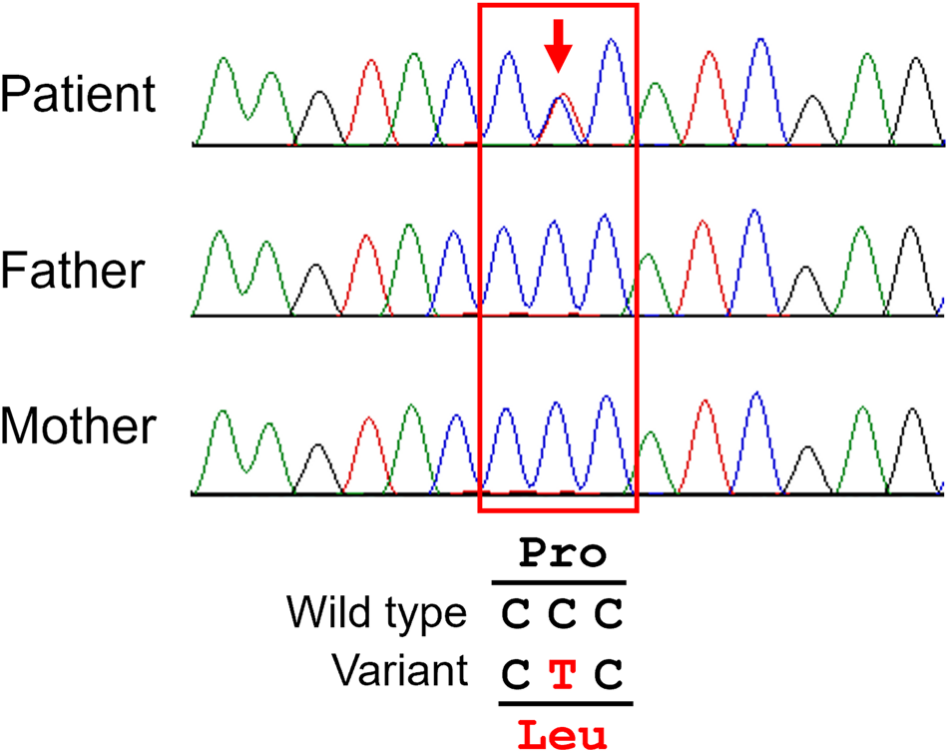
Sanger sequencing confirms missense variant (c.209C>T p.Pro70Leu) in the *ACTB* gene in the proband, but not in either parent.

BWCFF is a rare autosomal dominant disorder characterized by distinct craniofacial features, intellectual disability and various congenital anomalies. In this case, a de novo pathogenic variant in the *ACTB* gene (NM_001101.5:c.209C>T p.(Pro70Leu)) was identified using WES, further supporting the role of *ACTB* variants in the pathogenesis of BWCFF. This variant has not been reported in general population databases (for example, gnomAD), and its pathogenicity is supported by a previously reported case^2^, ClinVar reports, its location in a functional domain, evolutionary conservation and computational predictions. The clinical presentation of this patient overlapped substantially with the known spectrum of BWCFF, including craniofacial dysmorphisms, hearing loss, coloboma and developmental delay.

The present case exhibits a mild form of BWCFF, characterized by mild facial features, mild developmental delay and the absence of lissencephaly. Reports on the phenotype associated with the p.Pro70Leu variant are limited. One case harboring the p.Pro70Leu variant—reported by Verloes et al.^2^ (originally described by Bitton et al.^7^)—also exhibited a mild form of BWCFF without lissencephaly. In another case, the detailed phenotype was not fully elucidated because the subject was a terminated fetus; however, lissencephaly was not observed^5^. Similarly, the phenotype associated with p.Pro70Ala was also a mild form of BWCFF^8^. The residue Pro70 is located within the helix–loop (Subdomain1, SD1), which plays a key role in actin filament dynamics, specifically actin polymerization and depolymerization^8^. Proline possesses a cyclic structure, conferring rigidity to the polypeptide chain^9^. By contrast, leucine is a hydrophobic, linear amino acid with greater flexibility^10^. The substitution of proline with leucine may reduce local rigidity in the H-loop, potentially leading to excessive structural flexibility. Other pathogenic variants in SD1, including two cases with p.Gly74Ser and one case with p.Ile75Thr, were reported by Veloes et al.^2^. Among them, detailed symptoms were reported for only one case with p.Gly74Ser, which has been associated with severe BWCFF phenotypes, including severe intellectual disability and frontal pachygyria^11^. Glycine possesses an extremely flexible structure, maintaining the mobility of loops and turns^12^. However, its substitution with serine increases hydrogen bonding, leading to increased loop rigidity, which may result in ATP-binding abnormalities and impaired cell motility^13^. Similarly, the most common recurrent missense mutation at residue 196 (6 with Arg196Cys and 8 with Arg196His)^2,3^, is located within the loop structure (SD4) that is believed to facilitate actin alignment during the polymerization process. All the patients with Arg196 residue variants presented lissencephaly^3^. The substitution of positively charged arginine with cysteine or histidine leads to the loss of electrostatic stabilization, weakening actin monomer interactions and reducing filament stability. These changes indirectly affect ATP binding, impair ATP hydrolysis and disrupt actin filament polymerization-depolymerization dynamics, resulting in cell migration defects, neurodevelopmental abnormalities and morphological anomalies. In summary, the impact of the p.Pro70Leu is weaker compared with other pathogenic variants (for example, p.Gly74Ser and Arg196Cys) for the following reasons: (1) the absence of charge alteration results in minimal effects on ATP binding and filament stability; (2) while proline’s rigidity is reduced, leucine’s hydrophobicity helps maintain structural integrity; and (3) this variant does not substantially impact actin filament interactions, thus preserving relative filament stability.

This report contributes to the growing knowledge of *ACTB*-related phenotypes and adds evidence for the pathogenicity of the p.Pro70Leu variant. However, the limited availability of functional studies on this specific variant restricts a comprehensive understanding of its biological impact. Recently, induced pluripotent stem cells from a BWCFF with *ACTB* variant were established^14^. Future research, including cellular and animal models, could elucidate the precise molecular mechanisms by which *ACTB* variants disrupt normal development^3,15^.

In conclusion, this report presents a mildly affected case of BWCFF associated with the heterozygous p.Pro70Leu variant of *ACTB*. The phenotype in our patient—including coloboma, mild developmental delay and the absence of lissencephaly—closely resembles that observed in previously reported cases in the different population with the same variant, suggesting a genotype–phenotype correlation in this syndrome. Further investigations using cellular and animal models will be crucial to elucidate the precise molecular mechanisms underlying ACTB-related pathogenesis and to deepen our understanding of the phenotypic variability in BWCFF.

## HGV Database

The relevant data from this Data Report are hosted at the Human Genome Variation Database at 10.6084/m9.figshare.hgv.3524
